# Efficacy of dietary modifications and mucosal protectors in the treatment of laryngopharyngeal reflux: a multicenter study

**DOI:** 10.3389/fmed.2025.1488323

**Published:** 2025-01-27

**Authors:** Matteo Gelardi, Rossana Giancaspro, Christian Fiorentino, Stefano Patruno, Jacopo Marroni, Alessandro D’Avino, Letizia Neri, Michele Cassano, Giampiero Neri

**Affiliations:** ^1^Unit of Otolaryngology, Department of Clinical and Experimental Medicine, University of Foggia, Foggia, Italy; ^2^Neurosciences Imaging and Clinical Sciences Department, University of Chieti-Pescara, Chieti, Italy; ^3^Department of Medical Sciences, IDI-IRCCS, Rome, Italy; ^4^Department of Otolayngology, University of Insubria, Varese, VA, Italy

**Keywords:** laryngopharyngeal reflux, dietary modifications, mucosal protectors, pepsin, pep test

## Abstract

**Background:**

Laryngopharyngeal reflux (LPR) is defined as an extraesophageal reflux of gastroduodenal contents to the laryngopharynx, affecting the upper aerodigestive tract. There is currently no standardized treatment protocol for LPR. The use of proton pump inhibitors (PPIs) is widely established in common practice and derives from the standard approach of using PPIs to treat patients with gastroesophageal reflux disease (GERD). However, as PPIs may not be effective on all types of reflux, the aim of our study was to evaluate the effectiveness of dietary changes and mucosal protectants, alone or in combination, in LPR treatment.

**Methods:**

This multicenter randomized controlled trial included 48 patients divided into three groups: dietary modifications only, mucosal protectors only, and a combination of both. The patients’ responses were assessed over 1 month using the Reflux Symptom Index (RSI) and Reflux Finding Score (RFS), along with measurements of salivary and nasal pepsin concentration and rhinomanometry.

**Results:**

Significant improvements were observed in RSI and RFS scores across all groups. The group receiving combined dietary modifications and mucosal protectors showed the most substantial benefits. Additionally, a notable reduction in salivary and nasal pepsin concentrations and nasal resistances was observed, particularly in patients combining dietary modifications and mucosal protectors.

**Conclusion:**

The study showed that combined dietary modifications and mucosal protects strategies effectively manage LPR symptoms, offering a potential therapeutic approach.

## Introduction

Laryngopharyngeal reflux (LPR) is defined as extra-esophageal reflux of gastroduodenal content to the laryngopharynx, affecting the upper aerodigestive tract. Although LPR shares similar etiopathogenetic mechanisms with gastroesophageal reflux disease (GERD), in which gastric acid rises up the esophagus, LPR and GERD can be considered distinct entities, such that many patients with LPR-related symptoms have no GERD-associated symptoms (1). Indeed, LPR is not limited to a gastroenterological mechanism but also to biomechanical dysfunction and laryngeal hyperactivity (2).

Due to the multifactorial etiology, LPR-associated symptoms embrace a wide variety of clinical manifestations, such as morning hoarseness and nocturnal cough, chronic repetitive throat clearing, throat pain, excessive mucus production, dysphagia and foreign body sensation in the pharynx (3, 4). This symptoms are the expression of a series of morphological changes in the upper aerodigestive region, which are typical of the LPR, including supraglottic edema and erythema, cobbling of the posterior pharyngeal wall, vocal cord ulcers, interarytenoid changes, medial arytenoids wall edema and vocal cord granulomas (5).

LPR has emerged as a significant social burden in recent years, with studies indicating that up to 30% of otorhinolaryngology outpatient patients are affected by this condition (6).

Currently, there is no standardized diagnosis or treatment protocol. The assessment and examination of patients with laryngopharyngeal reflux (LPR) symptoms combines a comprehensive history and careful clinical evaluation with endoscopic findings, esophageal impedance-pH monitoring, salivary PEP test and/or questionnaires, such as the Reflux Symptom Index (RSI). Treatment options include lifestyle and dietary modifications, medical therapies, and, in some specialized centers, surgical interventions to address the various symptoms associated with the condition (7). As regards medical therapies, the use of proton pump inhibitors (PPIs) is widely established in common practice for patients suffering from LPR. This practice stems from the standard approach of using PPIs to treat patients with GERD. However, PPIs may not completely inhibit all types of reflux and are associated with adverse effects (8). Mucosal protectors, on the contrary, form a viscous mechanical barrier on the surface of stomach contents, thereby preventing or minimizing reflux of gastroduodenal contents, whether acid or non-acid.

Based on this background, the aim of our study was to evaluate the to assess the efficacy of dietary changes and mucosal protectors, either alone or in combination, in the treatment LPR.

## Materials and methods

### Study subjects

We conducted a multicenter, randomized, open-label study to evaluate and compare the efficacy of lifestyle modifications and mucosal protectors, either alone or in combination, in treating LPR.

Patients were recruited from October 2023 to April 2024 at the Departments of Otolaryngology of Chieti and Foggia University Hospitals.

Inclusion criteria were arranged as follows: age range: 20–72; genders: both; pathology: LPR (RSI > 13, RFS > 7); symptoms: at least weekly symptoms of LPR within a month prior to the start of the study.

Exclusion criteria were as follows: comorbidities: esophageal varix, Barrett’s esophagus, peptic ulcer, gastrointestinal bleeding, Zollinger–Ellison syndrome, or malignancy; previous surgeries: gastrointestinal operation, such as esophagectomy or gastrectomy; acute rhinitis; allergies: allergy to any of the drugs used in the study; personal therapy: histamine−2 receptor antagonists, PPIs, anticholinergic drugs, gastrin receptor antagonists, protective factor enhancers, gastric mucosal protective agents within 4 weeks of patient recruitment.

### Study protocol

**Figure 1:**
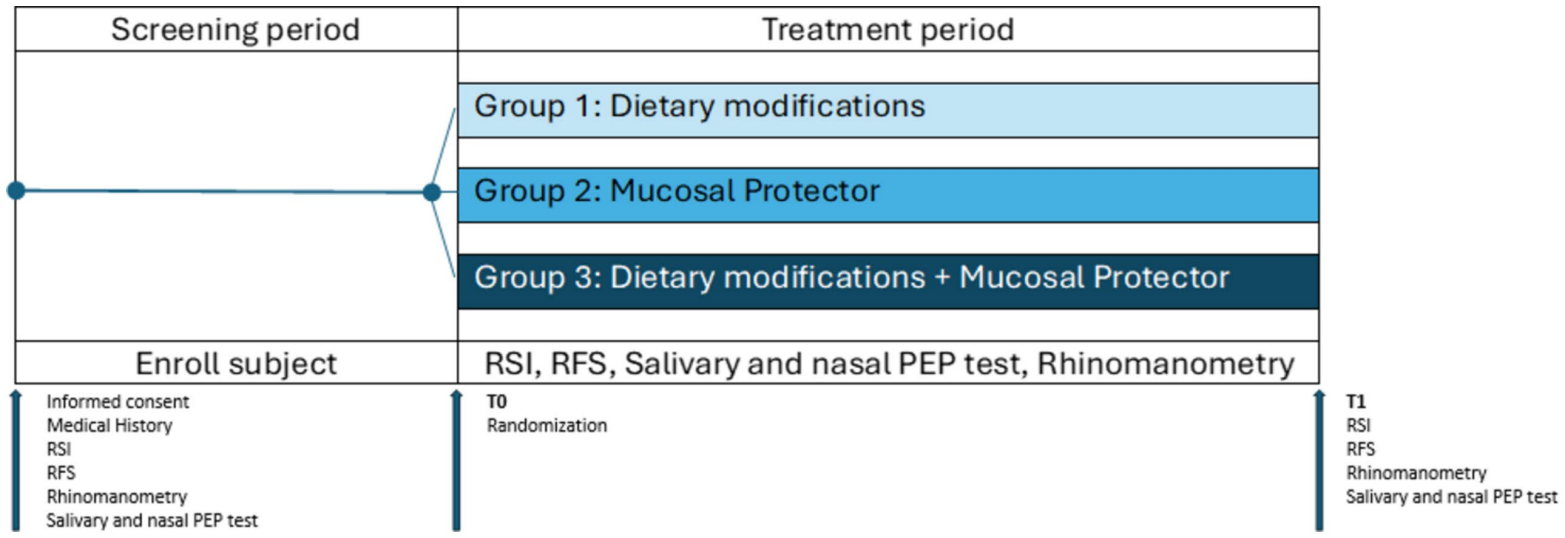
Study protocol. During the screening period, patients underwent medical history collection, clinical examination, and endoscopy of the upper aerodigestive tract, as well as PEP test and rhinomanometry, as screening tests. At T0, patients deemed eligible for the study were randomly assigned to the three groups: group 1, dietary modifications; group 2, mucosal protector; group 3, dietary modifications + mucosal protector. At T1, after 1 month, patients underwent the same evaluations performed during the screening phase to assess changes resulting from the proposed treatments (mucosal protector and dietary modifications, alone or in combination).

The subjects who participated in the clinical study firstly underwent careful medical history collection, clinical examination and endoscopy of the upper aerodigestive tract as screening tests. Salivary and nasal PEP test and rhinomanometry were performed on all patients. Forty-eight eligible patients were sequentially assigned into 3 groups in randomly: patients of Group 1 performed only dietary modifications; patients of Group 2 received mucosal protectors twice a day; patients of Group 3 associated dietary changes with mucosal protectors. The response to the different therapeutic approaches was evaluated after 1 month (Figure 1).

### Dietary modifications

**Figure 2:**
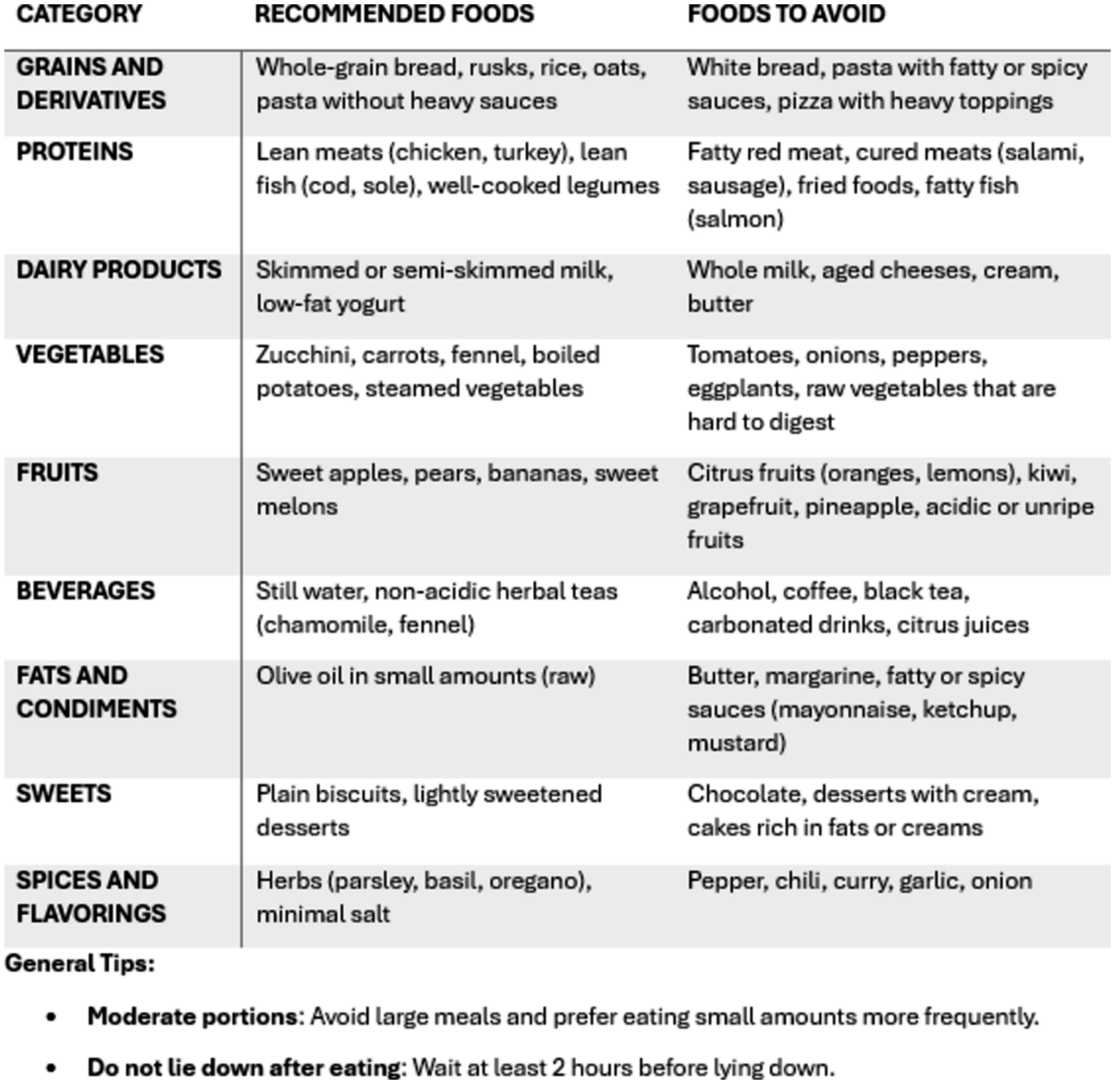
The list of recommended foods and the ones to avoid provided to patients.

Patients were instructed to follow low-fat, low-quick-release sugar, high-protein, alkaline, and plant-based diet. They were advised to limit their consumption of coffee, alcohol, tea, carbonated drinks, chocolates, spicy and fried foods, tomatoes, citrus fruits, onions and high-fiber vegetables (9, 10). To guide them in dietary modifications, they were provided with a booklet containing a list of foods to avoid or suggested, divided into categories (Figure 2).

### Mucosal protector

Patients in Group 2 and Group 3 took a 20 mL oral dose of a mucosal protector (Estorial®) 20 min after both lunch and dinner. This anti-acid anti-reflux medical device contains both magaldrate and alginate. Furthermore, among the components of Estorial®, there is M-ADESYL®, which is a patented mucoadhesive and film-forming composition based on low molecular weight hyaluronic acid, xanthan gum and plasdone K90 which adheres to the surface of the mucosa.

### Study measurements

The primary outcome was change in RSI scores. RSI is the most widely used type of symptom evaluation, and its validity and reliability are widely recognized around the globe. It is a nine-item self-reported questionnaire whereby a patient is asked to rate (0, no impact to 5, maximum impact) LPR-associated symptoms and how they have impacted their lives over the preceding month (Table 1). The maximum score is 45; a score of greater than 13 is considered a reliable diagnostic indicator for LPR (11).

**Table 1:** Reflux symptom index.

Within the last month, how did the following problems affect you?	0 = no problem 5 = severe problem					
Hoarseness or a problem with the voice	0	1	2	3	4	5
Cleaning you throat	0	1	2	3	4	5
Excess throat mucus or postnasal drip	0	1	2	3	4	5
Difficulty swallowing food, liquids, or pills	0	1	2	3	4	5
Coughing after you ate or after lying down	0	1	2	3	4	5
Breathing difficulties or choking episodes	0	1	2	3	4	5
Troublesome or annoying cough	0	1	2	3	4	5
Sensations of something sticking in your throat or a lump in your throat	0	1	2	3	4	5
Heartburn, chest pain, indigestion, or stomach acid coming up	0	1	2	3	4	5
Total						

A secondary outcome was change in RFS score, comprising an eight-item index, designed to score patients based on endoscopic findings; scores range from 0 to 26, with a score greater than 7 considered to be diagnostic for LPR (Table 2). RFS was blinded evaluated by and expert endoscopist using a Storz HD Video Rhino-Laryngoscope, 3.7 mm diameter.

**Table 2:** Reflux finding score.

Finding	Score
Subglottic edema	2 = present 0 = absent
Ventricular obliteration	2 = partial 4 = complete
Erythema/hyperemia	2 = arytenoids only 4 = diffuse
Vocal cord edema	1 = mild 2 = moderate 3 = severe 4 = polypoid
Diffuse laryngeal edema	1 = mild 2 = moderate 3 = severe 4 = obstructing
Posterior commissure hypertrophy	1 = mild 2 = moderate 3 = severe 4 = obstructing
Granuloma/granulation	2 = present 0 = absent
Thick endolaryngeal mucus/other	2 = present0 = absent
Total	

Additional measurements were basal nasal airflow resistance, measured by rhinomanometry, and salivary and nasal pepsin concentration, measured by PEP test. The pepsin concentration levels were accurately measured using an electronic reader, with a detection limit of 16 ng/mL and a quantitation range between 25 ng/mL and 500 ng/mL. For nasal PEP test, pepsin concentration was evaluated in the nasal lavage fluid using Rinowash.

### Ethics statement

This study was reviewed by the Institutional Review Board of Chieti University Hospital. All procedures performed in this study involving human participants were in accordance with the 1964 Declaration of Helsinki and its later amendments or comparable ethical standards. Informed consent was submitted by all subjects when they were enrolled.

### Statistics

Descriptive data were referred to as frequencies, medians and IQR. After normality tests and verification of the homogeneity of the groups, the following tests were chosen to evaluate the outcomes: Wilcoxon test to analyze the differences between T0 and T1 of the individual groups; Kruskal-Wallis test to analyze variations in means between groups; Multivariable linear regression models adjusted for the variables: Age, sex and BMI to find predictors of improvement in outcomes: Pep test (nasal and salivary), RSI, RSF and rhinomanometry. Statistical significance was defined as a *p* ≤ 0.05.

## Results

We recruited a total of 51 patients, randomly divided into the three groups (17 patients for each group). Of these, 3 did not comply with the instructions given at T0. The final study sample was 48 people (Group 1, *n* = 15; Group 2, *n* = 17; Group 3, *n* = 16), of which 19 (39.6%) were males and 29 (60.4%) were females. Median age was 52 (IQR = 43–64.5%). Median BMI was 24.8 (IQR = 23–28.7%). At time 0 (T0), the groups were homogeneous not only for demographic characteristics but also for the other variables.

**Figure 3:**
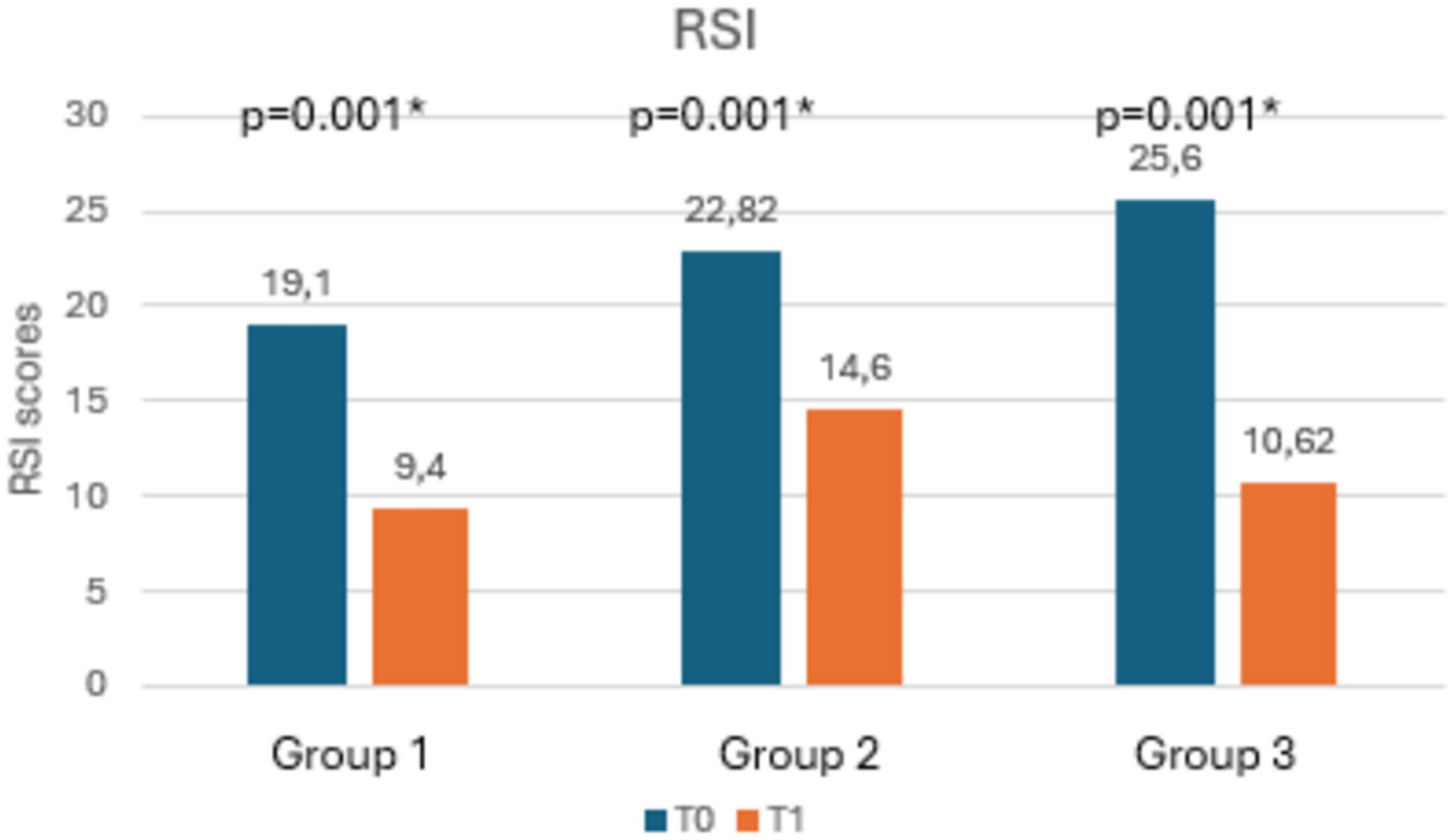
The histogram shows the variation in RSI scores at T0 and T1, comparing the three groups.

**Figure 4:**
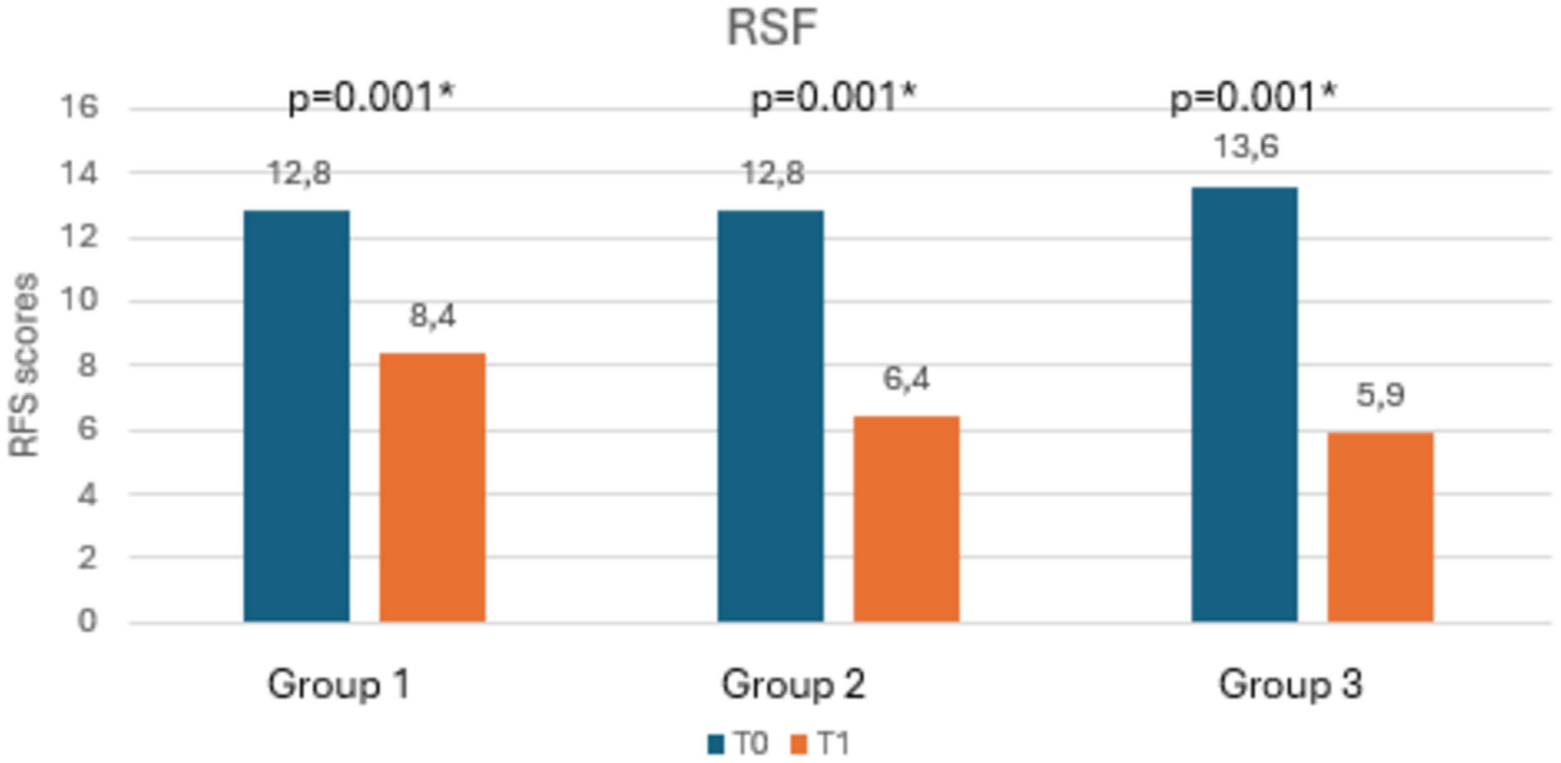
The histogram shows the variation in RFS scores at T0 and T1, comparing the three groups.

**Figure 5:**
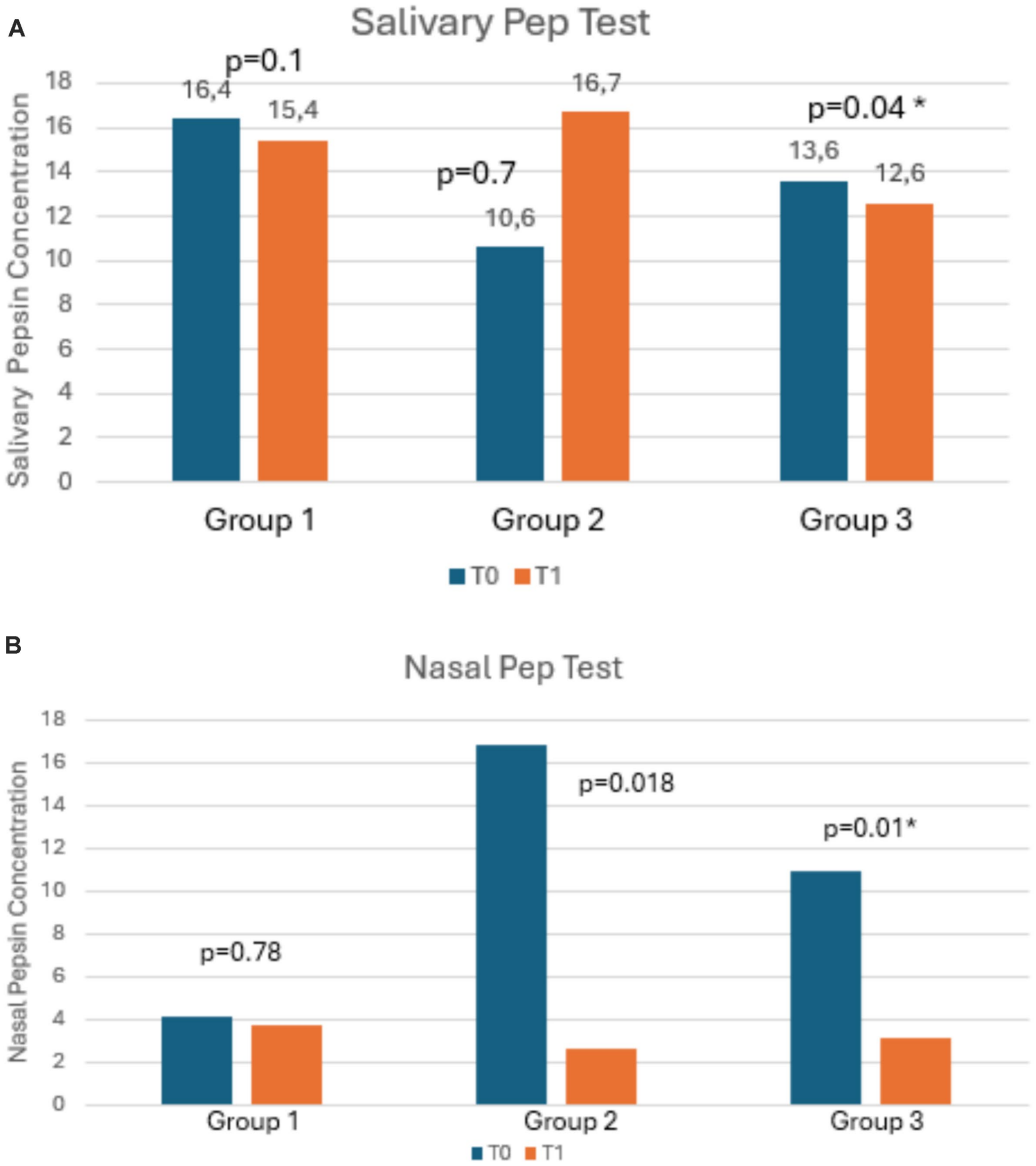
The histogram shows the variations in pepsin concentration at the salivary level **(A)** and nasal level **(B)** at T0 and T1, comparing the three groups.

**Figure 6:**
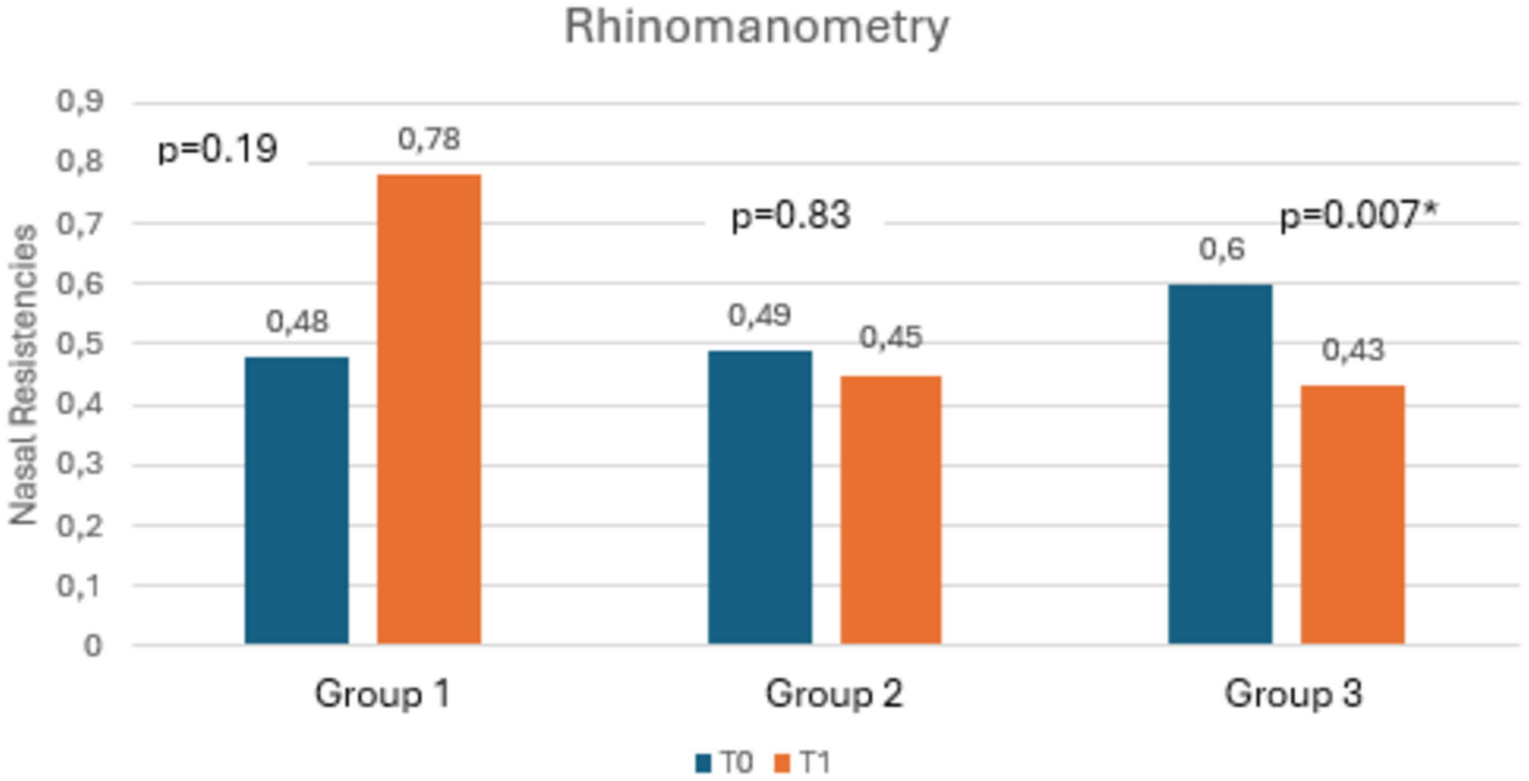
The histogram shows the variation in nasal resistances at T0 and T1, comparing the three groups.

Table 3 shows the mean scores of RSI, RFS, salivary and nasal PEP test, and rhinomanometry at T0 and T1, along with the statistical significances. Notably, as shown in Figures 3, 4, both RSI and RFS scores significantly decreased in all study groups at T1. On the contrary, as shown in Figures 5A,B, nasal pepsin concentrations statistically decreased in Group 2 and 3, while salivary pepsin concentrations significantly decreased only in Group 3 at T1. As regards rhinomanometry, nasal resistance statistically decreased after 1 month of treatment only in group 3 (Figure 6).

**Table 3:** Comparison of RSI, RFS, rhinomanometry, and nasal and salivary PEP test values at T0 and T1 in Group 1 (dietary modifications), Group 2 (mucosal protector), and Group 3 (dietary modifications + mucosal protector).

	T0	T1	*p* value
Group 1			
RSI, average	19.1	9.4	0.001*
RFS, average	12.8	8.4	0.001*
Nasal PEP test, average	4.1	3.7	0.78
Salivary PEP test, average	16.4	15.4	0.10
Rhinomanometry, average	0.48	0.78	0.19
Group 2			
RSI, average	22.82	14.6	<0.001*
RFS, average	12.82	6.41	<0.001*
Nasal PEP test, average	16.8	2.6	0.018*
Salivary PEP test, average	10.6	16.7	0.71
Rhinomanometry, average	0.49	0.45	0.83
Group 3			
RSI, average	25.6	10.6	<0.001*
RFS, average	13.6	5.9	<0.001*
Nasal PEP test naso, average	10.9	3.1	0.01*
Salivary PEP test, average	13.16	12.6	0.04*
Rhinomanometry, average	0.6	0.43	0.007*

The symbol * means statistically significant.

**Figure 7:**
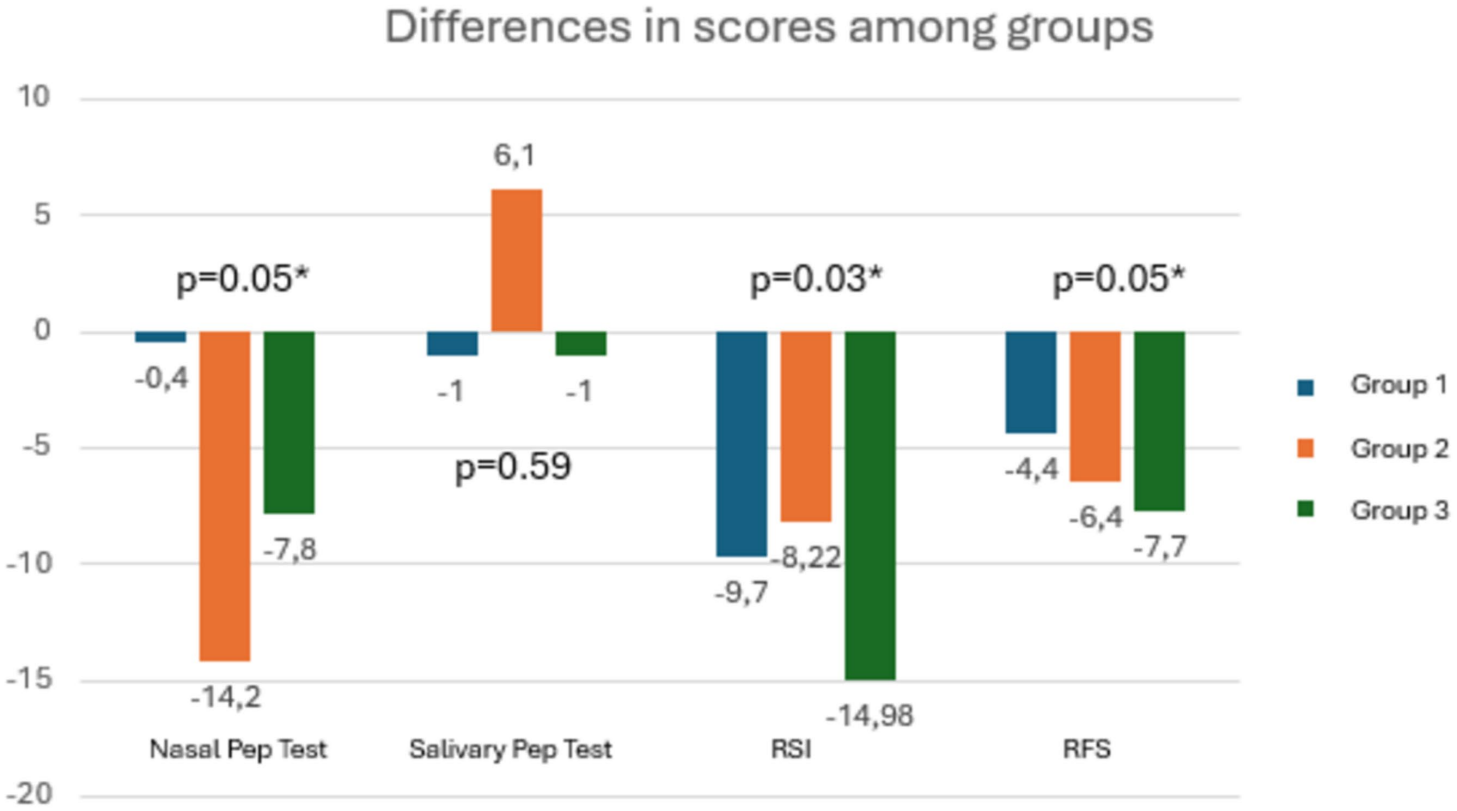
The graph shows that for all outcomes, patients in Group 3 demonstrated significantly greater improvement.

Analyzing the differences between groups in terms of changes in the means of clinical outcomes at T1, patients in Group 3 improved significantly more than the other two groups in terms of RSI and RSF scores and nasal PEP test (Figure 7). U-Mann Whitney test was performed to compare each couple of group in details (Table 4).

**Table 4:** Comparison among groups *p*-values of U-Mann Whitney test.

Groups	Pep test nasal	Pep test saliva	RSI	RSF
Dieta vs. Estorial	0.048*	0.89	0.75	0.09
Dieta vs. Estorial+Dieta	0.013*	0.31	0.14	0.08
Estorial vs. Estorial+Dieta	0.78	0.40	0.02*	0.53

The symbol * means statistically significant.

Moreover, the multivariable linear regression analysis showed that the mucosal protector appeared to be the only independent predictor of reduction in nasal Pep test and RFS. The same result from the mucosal protector, identified as an independent predictor of better outcomes in rhinomanometry and RSF values, was confirmed by replacing the regression analysis with models adjusted for age, sex, and BMI (Table 5).

**Table 5:** Estorial has been identified as an independent predictor of better outcomes in rhinomanometry and RSF values.

A. Dependent variable: RFS								
Model		Non standardized coefficients		Standardized coefficients	*t*	*P* value	95.0% Confidence interval for B	
		B	Error std.	Beta			Lower limit	Upper limit
1	Mucosal protector	−2.148*	1.041*	−0.305*	−2.064*	0.045*	−4.247*	−0.049*
	Sex	0.381	0.959	0.057	0.397	0.693	−1.553	2.315
	Age	−0.016	0.033	−0.075	−0.488	0.628	−0.083	0.051
	BMI	−0.036	0.087	−0.062	−0.415	0.680	−0.212	0.140

B. Dependent variable: rhinomanometry								
Model		Non standardized coefficients		Standardized coefficients	*t*	*p* value	95.0% Confidence interval for B	
		B	Error std.	Beta			Lower limit	Upper limit
1	Mucosal protector	−0.592*	0.176*	−0.539*	−3.357*	0.003*	−0.955*	−0.229*
	Sex	−0.216	0.162	−0.208	−1.330	0.196	−0.550	0.119
	Age	0.018*	0.005*	0.573*	3.441*	0.002*	0.007*	0.029*
	BMI	−0.008	0.013	−0.102	−0.648	0.523	−0.035	0.018

Models adjusted for age, sex and BMI. The symbol * means statistically significant.

## Discussion

For at least four decades, ENT literature has described the existence of gastric reflux with extradigestive laryngeal manifestations. However, the current conceptualization of LPR as a distinct nosological entity dates back only to the mid-1990s (12).

Nowadays LPR is an increasingly well-known entity and considerable effort has been made to understand its pathophysiological basis. The etiology of LPR is multifactorial and includes upper esophageal sphincter dysfunction, time of exposure to refluxed material, and level of tissue sensitivity. In particular, it is now known that the laryngeal epithelium is more sensitive to reflux than the esophageal one, so much so that only 3 episodes of LPR per week with a pH lower than 4 would be enough to cause damage at the laryngeal level. In the contrary GERD requires approximately 50 episodes per week to produce some degree of esophageal damage (13). The greater epithelial sensitivity could be explained by the presence of both low pH and pepsin at the laryngeal level, that in turn would cause an increase in the production of stress proteins.

Despite the undeniable progress in the knowledge of this disease, treatment remains challenging, since some patients, particularly those with non-acid or mixed LPR, do not fully benefit from PPIs, which aren’t able to completely prevent pepsin injury. Indeed, pepsin has been shown to be the most aggressive factor in both acid and non-acid LPR (14). Recent studies have suggested that although pepsin is reactivated by an acidic environment, a neutral pH environment in the laryngopharynx does not completely protect the mucosa from the inflammatory effects of pepsin, as the latter enzyme can reactivate within the lower pH intracellular enviroments (15, 16). Moreover, since pepsin survives for hours in the laryngopharynx after a LPR event, it can be reactivated by dietary acids (17).

An ideal and comprehensive therapeutic approach to LPR should include not only dietary changes and PPIs, but also medical devices known as mucosal protectors. The latter are barrier-forming agents that create a protective film on the mucosa of the upper aerodigestive tract and of the esophagus, which helps prevent mucosal damage. Additionally, they might physically obstruct the reflux of gastroduodenal content to the laryngopharynx through a “floating raft” effect (18).

Indeed, although PPIs have been considered as primary medical treatment of LPR for a long time, recent literature has increasingly focused on cost and side effects of PPIs, especially as PPI prescriptions might be unnecessary if acid reflux is not the cause of patient complaints. Moreover, meta-analyses have demonstrated that PPIs may be no better than placebo in treating presumed LPR disease (19). On the contrary, studies have shown that alginate or magaldrate products, which both act on weakly acidic or nonacidic reflux events, may be effective in LPR when used alone or when used in combination with PPIs (20).

Despite emerging evidence on the overall efficacy of mucosal protectors, suggesting that the alginate raft is intended to physically limit reflux of gastric contents proximally and that alginates can potentially be an effective barrier agent in LPR of any pH, studies on the roles alginates and magaldrates in the treatment of LPR remain scarce and limited (21).

Given this context, we aimed to investigate the efficacy of dietary changes and mucosal protectors, either alone or in combination, in the treatment LPR, evaluating RSI and RFS score, rhinomanometry, and salivary and nasal pepsin concentration.

An interesting finding from the study, albeit not statistically significant, was the loss of patients from Groups 1 and 3, which involved dietary modifications, and none from group 2, which did not require dietary changes. In Italy, the Mediterranean diet is widely adopted and, although healthy, it is not always in line with the recommendations for managing reflux. Therefore, patients accustomed to adopting a Mediterranean diet were less likely to adopt a low-fat, low-quick-release sugar, high-protein, alkaline, and plant-based diet. On the contrary, they were more likely to assume a mucosal protector with the view that this would allow them not to change their eating habits and, at the same time, obtain clinical-symptomatic benefits.

As regards the primary outcome, a statistically significant reduction in RSI scores was found in all groups. The same result was found for the secondary outcome, as all groups showed a statistically significant reduction in RFS scores. These data are in line with current literature, since dietary modifications and the use of alginates or magaldrates had already been shown to be able to reduce LPR-related symptoms as well as to improve endoscopic findings (17).

To date, no study has evaluated the effects of mucosal protector and dietary modifications on salivary and nasal pepsin concentrations as well as on nasal resistances.

As shown in the results, patients who adopted solely dietary changes did not found a significant reduction in pepsin concentrations, both in salivary and nasal PEP test, neither in rhinomanometry. Patients receiving Estorial® alone shown a reduction in nasal pepsin concentration. The observed reduction in pepsin concentrations at the nasal level, before the salivary one, could be justified by the protective effect of the mucosal protector, which decreased the frequency and the extent of gastric reflux, lowering pepsin which reaches higher anatomical levels. As a result, nasal pepsin levels decreased first. However, the protective effect should progressively extend to lower anatomical levels, leading to a subsequent decrease in pepsin concentration at the pharyngeal level.

In this context, it is not clear why we found increased concentrations of salivary pepsin at T1 in this group of patients, although not statistically significant. Probably, in part this result depends on the fact that the acidic food environment may have kept the activated levels of pepsin at the pharyngeal level high (22). However, we believe that probably by prolonging the therapy with the mucosal protector alone we would have also found a reduction in salivary pepsin.

Only the group of patients who adopted the combination of dietary measures and Estorial® showed an improvement of all outcomes, including salivary and nasal PEP test and rhinomanometry. The combination of the two treatments could, in fact, not only reduce LPR episodes but also reduce the reactivation of pepsin at the pharyngolaryngeal level. Indeed, the mucosal protector adopted contains both magaldrate and alginate, the former neutralizes excess gastric acidity, the latter blocks the rise of the acidic contents of the stomach. These components are associated with a mucoadhesive mixture based on hyaluronic acid which adheres to the mucosa of the upper aerodigestive tracts, creating protective layer that facilitates healing and regeneration of the mucosal membrane (23).

By limiting gastric hyperacidity, hindering the passage of gastroduodenal contents toward the esophagus and preserving the mucosa from acid irritation, Estorial® could help protect the mucosa and consequently improve not only the symptoms but also lead to a reduction or resolution of morphological changes within the upper aerodigestive region. The association with dietary changes could improve these effects, also reducing the concentration of pepsin at the salivary and nasal levels and nasal resistances, measured through rhinomanometry. As a matter of fact, LPR is associated with several otolaryngological conditions, including rhinitis, sleep apnea and chronic otitis media (24, 25). Particularly, LPR and GERD are considered important causes of non-allergic rhinitis with neutrophils, as exhaling hydrochloric acid and its contact with the nasal mucosa attracts neutrophils, first response inflammatory cells (26). The permanence of these cells and the release of chemical mediators such as neutrophilic elastase leads to the formation of free radicals and to mucosal damage, which results in “vasomotor” symptoms, such as sneezing, nasal congestion and seromucous rhinorrhea (27). It is therefore not surprising that good control of LPR leads to a reduction of acid and pepsin in at the nasal mucosa level, with a consequent reduction of the immunophlogistic infiltrate and nasal resistances. Indeed, it is worth mentioning that although all groups showed a significant reduction in RSI and RFS, the group that adopted both dietary measures and Estorial® improved significantly more than the other two groups in terms of nasal PEP test, RSI and RSF scores.

It should also be underlined that Estorial® was found to be the only independent predictor of reduction in nasal PEP test and RFS. Therefore, despite the small sample size, Estorial® proved to be a valid medical device for reducing sings and symptoms of LPR patient; moreover, when associated with an adequate non-acid diet, it also showed an add-on therapy effect with significant improvements in nasal resistances.

Further studies are necessary not only to confirm the beneficial effect of Estorial® as an add-on therapy for LPR, but also to establish whether the combination of diet and anti-acid anti-reflux mucosal protectors can replace the use of PPIs in LPR patients. In this context, with a view to guaranteeing patients effective treatments burdened by the least number of side effects, defining the most suitable diagnostic-therapeutic path for patient suffering from LPR appears of utmost importance.

## Data Availability

The original contributions presented in the study are included in the article/supplementary material, further inquiries can be directed to the corresponding author.
